# The potential radiosensitization target PFKFB3 is related to response to radiotherapy in SweBCG91RT: a randomized clinical trial with long-term follow-up

**DOI:** 10.1186/s12885-025-13703-1

**Published:** 2025-02-28

**Authors:** Moa Egelberg, Tommaso De Marchi, Niklas Schultz, Lena Tran, Per Karlsson, Erik Holmberg, Gyula Pekar, Fredrika Killander, Emma Niméus

**Affiliations:** 1 Division of Surgery, Department of Clinical Sciences Lund, Faculty of Medicine, Lund University, BMC D13, Lund, 221 83 Sweden; 2 Division of Oncology and Pathology, Department of Clinical Sciences Lund, Faculty of Medicine, Lund University, Lund, Sweden; 3 Department of Radiology, Kristianstad Hospital, Kristianstad, Sweden; 4 Science for Life Laboratory, Department of Oncology-Pathology, Karolinska Institutet, Stockholm, Sweden; 5 Department of Oncology, Institute of Clinical Sciences, Sahlgrenska Academy, Sahlgrenska University Hospital, University of Gothenburg, Gothenburg, Sweden; 6 Department of Clinical Pathology and Cytology, Unilabs AB, Skaraborg Hospital, Skövde, Sweden; 7 Division of Oncology and Pathology, Department of Clinical Sciences Lund, Faculty of Medicine, Skåne University Hospital, Lund, Sweden; 8 Division of Surgery, Department of Clinical Sciences Lund, Faculty of Medicine, Skåne University Hospital, Lund, Sweden

**Keywords:** Breast cancer, PFKFB3, Radiotherapy, Radiosensitization, DNA repair

## Abstract

**Background:**

Several cancer types have increased PFKFB3, a glycolytic enzyme for which potent inhibitors have been found. Inhibition of PFKFB3 impairs DNA repair after irradiation of cancer cells, making it a possible radiosensitization target. The SweBCG91RT trial, in which breast cancer patients were randomized to postoperative radiotherapy or not, was used to investigate PFKFB3 as a clinical marker of sensitivity to adjuvant radiotherapy.

**Methods:**

Nuclear protein levels of PFKFB3 were assessed with immunohistochemistry in primary breast tumors (*n* = 970) and whole-cell RNA levels with microarray gene expression (*n* = 765). Multivariable competing risks regression analysis was employed for the effect of radiotherapy on incidence of ipsilateral breast tumor recurrence (IBTR), depending on PFKFB3 levels.

**Results:**

Tumors with high levels of nuclear protein and RNA had the largest effect on incidence of IBTR of adjuvant radiotherapy, however without evidence of an interaction. *PFKFB3* RNA correlated with subtype, as high levels were more common among the human epidermal growth factor receptor 2 (HER2) positive and Luminal A subtypes than Luminal B and triple negative tumors.

**Conclusion:**

High PFKFB3 is associated with a larger reduction of IBTR after radiotherapy but PFKFB3 cannot reliably be used as a predictive marker of sensitivity to adjuvant radiotherapy in breast cancer. *PFKFB3* expression differed with subtype, indicating that it may be a better marker among Luminal A and HER2 positive tumors, but this is yet to be investigated.

**Trial registration:**

The trial has been retrospectively registered at clinicaltrials.gov 2024-10-03 (NCT06637202).

**Supplementary Information:**

The online version contains supplementary material available at 10.1186/s12885-025-13703-1.

## Introduction

The use of adjuvant radiotherapy as a complement to breast conserving surgery is widely accepted to decrease the rate of locoregionally recurring cancer. It has been proven effective regardless of tumor size and subtype [1]. However, some patients develop recurrences in spite of radiotherapy and these tumors can be considered resistant to radiotherapy. Since radiotherapy is associated with risks such as skin reactions, cardiac toxicity, and pneumonitis [2], a biomarker predictive of radiotherapy sensitivity is needed.

PFKFB3 is a metabolic enzyme and a suggested cancer treatment target. Inhibition of PFKFB3 results in cytostatic effects and enhancement of chemotherapy and radiotherapy efficacy. PFKFB3 belongs to a family of 6-phosphofructo-2-kinase/fructose-2,6-bisphosphatases (PFKFBs), which are bifunctional, key-regulatory enzymes in glycolysis. The third enzyme in the family, PFKFB3, has shown to be abundant in several cancer types, including breast cancer [3–7]. Its transcription is increased by hypoxia-inducible factor 1α (HIF-1α) as a response to hypoxia [8] which is common in cancer cells due to rapid growth and inadequate blood supply. Potent inhibitors to the enzyme have been found and tested both in vitro and in mice [3, 9–12]. Inhibition of PFKFB3 in mouse models have shown to decrease glucose uptake and increase apoptosis in cancer cells [12]. In a human epidermal growth factor receptor 2 (HER2 positive) breast cancer mouse model, inhibition of PFKFB3 reduced glucose uptake as well as tumor growth [4], making it a promising treatment target in HER2 positive breast cancer. In addition to this, overexpression of PFKFB3 increases proliferation of cancer cells, without affecting glucose metabolism [13]. Thus, PFKFB3 has important functions in cancer cells, including glycolysis and proliferation.

Importantly, PFKFB3 has been identified as a regulator of the DNA double strand break repair homologous recombination (HR) [11]. Upon irradiation of cells, PFKFB3 relocated into nuclear DNA repair foci. Knockdown of PFKFB3 eliminated the recruitment of HR repair proteins (such as BRCA1 and RAD51) and reduced the HR repair activity. Both silencing and inhibition of PFKFB3 enhanced radiosensitivity, as cells showed delayed cell cycle progression upon irradiation and reduced long-term survival. Thus, PFKFB3 is a potential target for radiosensitization treatment, which is needed in the treatment of several cancer forms including breast cancer. However, the relationship between expression of PFKFB3 in non-treated breast cancer cells and radiotherapy has not been studied. The expression of PFKFB3 in untreated tumors may itself be a predictor of the clinical use of adjuvant radiotherapy. Further, more information regarding PFKFB3 in breast cancer is needed, to guide future potential inhibitory treatment.

To investigate this, we used a unique breast cancer material that specifically allows for the assessment of the effects of adjuvant radiotherapy on disease recurrence rates. SweBCG91RT is a large trial in which patients have been randomized to postoperative radiotherapy or not with long-term follow-up [14, 15]. The majority of the primary tumors have been included in a tissue microarray (TMA) [16] and microarray gene expression analysis has been performed [17]. Thus, the SweBCG91RT cohort is an extensive and well-suited sample set for the evaluation of potential immunohistochemical and gene expression markers and use of adjuvant radiotherapy in breast cancer.

The primary aim of this study was to assess PFKFB3 protein and RNA levels in relation to reduced incidence of ipsilateral breast tumor recurrence (IBTR) after adjuvant radiotherapy. To deepen the knowledge on PFKFB3 in breast cancer and to aid the potential use of future inhibitors, secondary aims were to study PFKFB3 levels in different breast cancer subtypes, to correlate it to clinicopathologically relevant factors, gene expression pathways and outcome.

## Materials and methods

### Study design

**Fig. 1:**
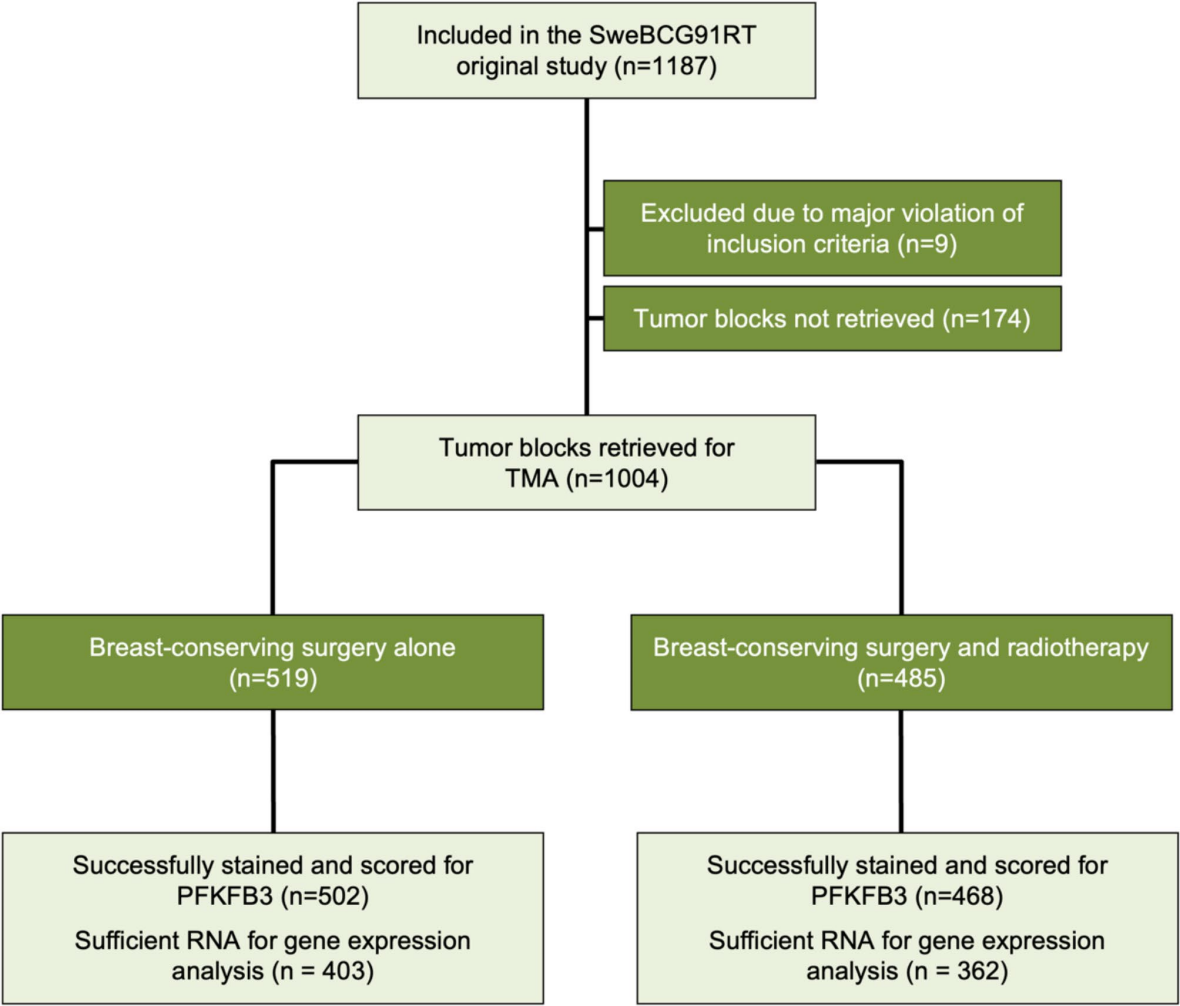
CONSORT diagram of SweBCG91RT and division of tumors in non-radiotherapy and radiotherapy-groups. TMA – tissue microarray

The formation and long-term follow-up of the original trial SweBCG91RT has been described before [14, 15]. In short, 1187 patients with early-stage breast cancer in Sweden were randomized to radiotherapy or not after breast-conserving surgery between 1991 and 1997, (Fig. 1). Patients had T1/T2N0M0 disease. All tumors were radically resected and thus had no tumor cells in the surgical margins. Radiotherapy was given to the whole remaining breast in 24–27 fractions (48–50 Gy in total). Apart from radiotherapy, limited adjuvant treatment was given. Patients were followed clinically for ten years and then via the mammography screening program until 74 years of age. Medical records were used to identify cases of recurring disease. The Swedish population register and the Swedish Cause of Death register, both with high reporting rates and accuracy [18, 19], were used to find deceased patients and their cause of death.

### TMA and immunohistochemistry

**Fig. 2:**
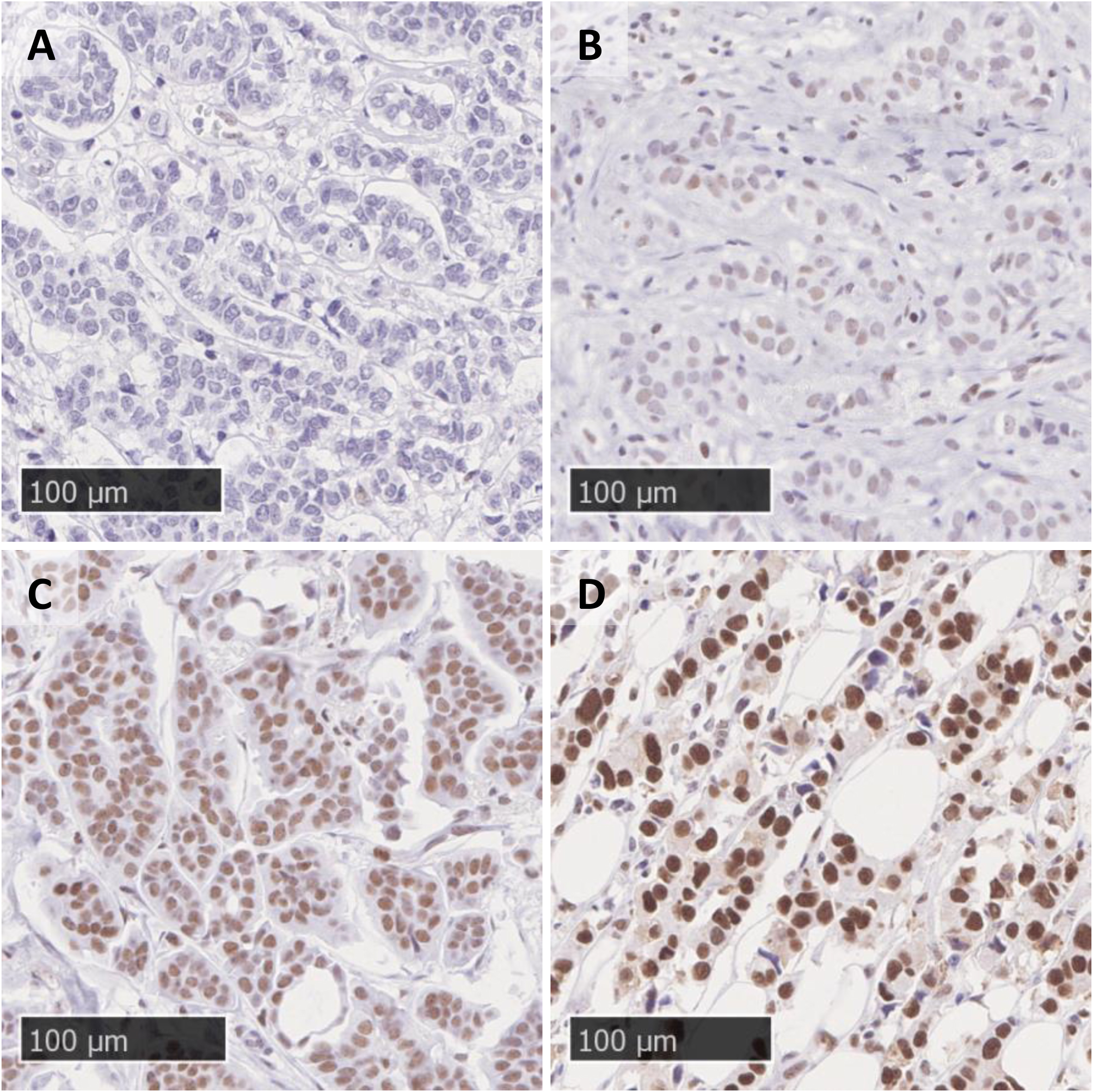
**A**-**D**. Different immunohistochemical staining intensities for nuclear PFKFB3 protein. (**A**) Negative, (**B**) Weak, (**C**) Moderate, and (**D**) Strong

As previously described [16], a TMA was constructed using all retrievable primary tumors (*n* = 1004) as well as IBTRs (*n* = 141). The non-available primary tumors were marginally smaller. Two 1.0 mm-cores were taken from representative areas of the formalin-fixed paraffin-embedded tumor material, marked by a board-certified pathologist. Evaluations of the markers used in clinical practice were performed; estrogen receptor (ER), progesterone receptor (PgR), Ki-67 and HER2. Using the guidelines from the St Gallen International Breast Cancer Conference (2013) Expert Panel [20], a cancer subtype was assigned to each tumor (Supplementary Table 1). Due to small group sizes, the luminal and non-luminal HER2 positive tumors were considered one group. Accordingly, the subtypes were as follows: 555 luminal A-like (ER+, PgR high, HER2-, Ki-67 low), 259 luminal B-like (PgR low and/or Ki-67 high, ER+, HER2-), 64 HER2 positive (HER2+, any ER/PgR/Ki-67), and 81 triple negative (ER-, PgR-, HER2-, any Ki67). Forty-five tumors had missing data in some or all of the variables and were not assigned any subtype. Histological grade was assessed on whole sections and according to Elston and Ellis’ Nottingham grading system [21] (missing for 46 tumors). For the current study, the TMAs were cut into 3–4 μm sections. Antigen retrieval was performed on a PT-LINK (Agilent Technologies, Santa Clara, CA, US) instrument using the Envision Flex Target Retrieval solution (pH 6, dilution 1:10) at 98 °C for 20 min. The slides were incubated for 30 min with the primary antibody against PFKFB3 (ab181861, clone EPR12594, Abcam, Cambridge, UK, dilution 1:500) using the instrument Autostainer Plus (Agilent Technologies). For visualization of the antigen-antibody complex the Envision Flex DAB detection kit K801021-2 (Agilent Technologies) was used. Scoring was performed by a breast-pathologist and the scoring criteria were based on a previous study [5]. Nuclear staining was assessed in terms of fraction of stained tumor cells; 0 (< 5%), 1 (5–25%), 2 (26–50%), 3 (51–75%), 4 (> 75%). Nuclear intensity of staining was scored as 0 (Negative), 1 (Weak), 2 (Moderate), or 3 (Strong), see Fig. 2. If the staining intensity was heterogenous, the most common intensity level was used. The nuclear fraction score and the nuclear intensity score were added into a combined histoscore with values from 0 to 7. A TMA core was excluded if it contained less than 50 tumor cells. If the two TMA cores from the same tumor showed different staining results, the one with the highest fraction of stained tumor cells was used. Cytoplasmatic staining was uncommon and not evaluated. Staining for biologically related markers (RAD51, geminin, micronuclei, γH2AX, p53, Ki67, HIF-1α, and pAkt) was performed and is described in the supplementary methods section.

### RNA expression

As previously described [17], RNA data was acquired for 765 primary tumors from 1.5 mm tumor tissue punches using RNeasy FFPE kit (Qiagen, Hilden, Germany). Tumors not included for gene expression analysis were marginally smaller and more likely to be of Nottingham grade 1 and Luminal A-like subtype [17]. With the Ovation FFPE WTA system (NuGEN, San Carlos, CA, US), cDNA was amplified, fragmented, and labeled using Encore Biotin Module (NuGEN) and hybridized to Gene-Chip Human Exon 1.0 ST Arrays. Before the data was delivered from Thermo Fisher Scientific (South San Francisco, CA, US) it underwent Single Channel Array Normalization [22]. Gene expression data is available at Gene Expression Omnibus (accession number GSE119295).

### PFKFB3 grouping

Tumors were divided into groups based on nuclear PFKFB3 protein levels and RNA levels. Four as equal sized groups as possible of different nuclear protein levels were created based on the distribution of histoscores among the primary tumors (Supplementary Fig. 1). This was done rather than according to the subgrouping in the previous article [5], as our tumors had higher PFKFB3 histoscores. Protein groups were thus: - (*n* = 210, histoscore 0–4), + (*n* = 183, histoscore 5), ++ (*n* = 314, histoscore 6), +++ (*n* = 263, histoscore 7). Based on RNA levels, tumors were separated into quartiles (Q): Q1 (*n* = 192), Q2 (*n* = 191), Q3 (*n* = 191), and Q4 (*n* = 191).

### Statistical analysis

Data analysis was performed in STATA (v18, College Station, TX, US) and R (v4.0.5, Vienna, Austria). Spearman’s rank correlation was used to test the correlation between protein and RNA levels. The PFKFB3 groups (protein and RNA) were compared to clinicopathological variables using the Kruskal Wallis test, the Pearson’s χ^2^ test, and the Fisher’s exact test where appropriate. In survival analysis, competing-risk regression was performed using the Fine and Gray method [23]. Subhazard ratios (SHRs) and 95% confidence intervals (CIs) are presented. For IBTR as first event, competing risks were other recurrences and death from any cause. To increase the proportion of true recurrences and limit new, ipsilateral breast tumors, the analysis was restricted to the first 10 years after randomization. For BCD, the competing risk was death from other causes and the cutoff point was set to 15 years after randomization. Separate analyses were performed for PFKFB3 protein and RNA groups. For each outcome, univariable regression analyses were performed with PFKFB3, age, radiotherapy, adjuvant systemic treatment, subtype, Nottingham grade, and tumor size as variables. To study radiobiology, radiotherapy grouping was based on given radiotherapy and not the intention to treat. In the multivariable analysis all significant variables out of the univariable analyses were employed in generating the regression model, while PFKFB3 and breast cancer subtype were kept regardless of significance. To investigate the relationship between PFKFB3 and incidence of IBTR after radiotherapy, an interaction term between PFKFB3 and radiotherapy was introduced in the multivariable IBTR analyses. To present the effect of radiotherapy on the incidence of IBTR in each PFKFB3 group, the reference group for PFKFB3 was changed. With the user-written command stcompet.ado in Stata [24], cumulative incidence functions were created and are presented for IBTR. The Wilcoxon rank-sum test was used to test the difference in PFKFB3 histoscore in 116 IBTRs compared to their paired primary tumor, depending on radiotherapy treatment. To test PFKFB3 as a prognostic marker, protein and RNA levels were tested in relation to IBTR and BCD. To explore the effect of PFKFB3 in different subtypes, the analysis was repeated using each subtype as reference variable. To test the proportional hazard assumption, time interaction with all covariates was performed in the regression analyses. The proportional hazard assumption was violated for the variables adjuvant systemic treatment (endocrine + chemotherapy) and Nottingham grade (3) in the radiotherapy efficacy analysis for protein. In the prognostic IBTR protein analysis it was violated for abovementioned variables and radiotherapy. In the prognostic IBTR RNA analysis it was violated for radiotherapy. In the prognostic BCD analyses, it was violated for subtype (triple negative) and Nottingham grade (3) in both protein and RNA analysis, and for PFKFB3 protein (+ and +++) in protein analysis. Thus, these SHRs should be considered as mean effects over the entire period. Differential expression analysis on RNA data between PFKFB3 protein groups was performed using limma (v3.46.0). To determine enriched pathways based on PFKFB3 groups Pathway enrichment analyses between conditions and cell models were performed using Gene Set Enrichment Analysis (GSEA) [25]. Settings were as follows: Hallmarks database (v5.2); permutation type: gene set; scoring: classic; metric: t test. Other parameters were kept to default settings. Correlations between PFKFB3 protein and associated markers were tested with the Spearman’s rank correlation using continuous histoscore or with χ^2^ test and categorical protein groups.

## Results

Out of 1004 primary tumors, 970 were successfully scored for nuclear PFKFB3 protein levels. RNA levels were available for 765 tumors. The correlation of histoscore between the two TMA cores for each tumor was strong (Spearman ρ = 0.79, *p* < 0.001). However, the correlation between nuclear PFKFB3 protein and RNA levels was weak (Spearman ρ = 0.37, *p* < 0.001), which may be due to the immunohistochemical staining assessing nuclear protein in tumor cells and the RNA measurement is whole-cell material from all cells in the tissue punch. Out of the 970 patients, 468 received adjuvant radiotherapy and 502 did not (Fig. 1). In the radiotherapy group, 31 (7%) also had systemic adjuvant therapy, of which five had chemotherapy (alone or in combination with endocrine therapy). In the no radiotherapy group, 49 patients (10%) received systemic adjuvant therapy out of which twelve had adjuvant chemotherapy.

### PFKFB3 protein and clinicopathological variables

When comparing commonly employed clinicopathological prognostic factors and PFKFB3 protein, strong evidence for an association between Nottingham grade and nuclear PFKFB3 protein groups was found (*p* = 0.002, Table 1). Few tumors among the highest group (+++) were of grade I (7% versus 16–19% among the other groups). No differences were seen in regards of patient age, tumor size, adjuvant systemic treatment, or subtype (Table 1 and Supplementary Table 2). In conclusion, we observed a positive association between nuclear PFKFB3 protein levels and tumor grade.

**Table 1:** Association between PFKFB3 protein and clinicopathological variables

	PFKFB3 protein				
	-	+	++	+++	*p*
	*n* = 210	*n* = 183	*n* = 314	*n* = 263	
**Age**					
Median (IQR), years	58 (52–66)	59 (51–66)	60 (50–66)	61 (51–68)	0.42
**Tumor size** ^a^					
Median (IQR), mm	12 (10–15)	12 (9–16)	12 (10-16.5)	12 (10–16)	0.74
**Nottingham grade** ^b^					
I	36 (18%)	28 (16%)	58 (19%)	18 (7%)	
II	107 (55%)	103 (58%)	185 (60%)	168 (65%)	
III	53 (27%)	46 (26%)	64 (21%)	71 (28%)	
Total	196 (100%)	177 (100%)	307 (100%)	257 (100%)	0.002
**Subtype** ^c^					
Luminal A-like	104 (52%)	110 (61%)	187 (61%)	146 (57%)	
Luminal B-like	62 (31%)	43 (24%)	81 (26%)	69 (27%)	
HER2 positive	12 (6%)	13 (7%)	15 (5%)	23 (9%)	
Triple negative	21 (11%)	14 (8%)	23 (8%)	20 (8%)	
Total	199 (100%)	180 (100%)	306 (100%)	258 (100%)	0.42

Number (%) if not stated otherwise. IQR – interquartile range, mm – millimeters, p – p-value, SD – standard deviation. ^a^ missing data for 6 patients, ^b^ missing data for 33 patients, ^c^ missing data for 27 patients

### PFKFB3 RNA and clinicopathological variables

There was moderate evidence for an association between *PFKFB3* RNA groups and subtype (*p* = 0.028, Table 2 and Supplementary Fig. 2). A stepwise increase in frequency of Luminal A-like tumors with increasing RNA levels was found. A positive association was also observed for HER2 positive tumors as the highest RNA group (Q4) had the highest frequency of HER2 positive tumors (10% vs. 5–8%), but this association was not consistent among all RNA groups. An inverse relationship between the frequency of Luminal B-like and triple negative tumors and *PFKFB3* RNA groups was observed. No association was found between *PFKFB3* RNA and the other clinicopathological variables (Table 2 and Supplementary Table 2). In conclusion, we observed varying *PFKFB3* RNA expression in different breast cancer subtypes.

**Table 2:** Association between *PFKFB3* RNA levels and clinicopathological variables

	PFKFB3 RNA				
	Q1	Q2	Q3	Q4	*p*
	*n* = 192	*n* = 191	*n* = 191	*n* = 191	
**Age**					
Mean (SD), years	58.5 (52–66)	57 (50–65)	60 (52–67)	61 (50–66)	0.52
**Tumor size** ^a^					
Median (IQR), mm	12 (10–17)	12 (10–16)	12 (9–15)	13 (10–16)	0.40
**Nottingham grade** ^b^					
I	36 (19%)	27 (14%)	21 (11%)	21 (11%)	
II	106 (56%)	116 (62%)	117 (62%)	118 (63%)	
III	48 (25%)	45 (24%)	51 (27%)	47 (25%)	
Total	190 (100%)	188 (100%)	189 (100%)	186 (100%)	0.33
**Subtype** ^c^					
Luminal A-like	93 (49%)	102 (54%)	112 (60%)	114 (60%)	
Luminal B-like	62 (33%)	54 (28%)	50 (27%)	50 (26%)	
HER2 positive	9 (5%)	16 (8%)	11 (6%)	18 (10%)	
Triple negative	25 (13%)	18 (9%)	15 (8%)	7 (4%)	
Total	189 (100%)	190 (100%)	188 (100%)	189 (100%)	0.028

Number (%) if not stated otherwise. IQR – interquartile range, mm – millimeters, p – p-value, SD – standard deviation. ^a^ missing data for 5 patients, ^b^ missing data for 12 patients, ^c^ missing data for 9 patients

### Effect of adjuvant radiotherapy on incidence of IBTR

**Fig. 3:**
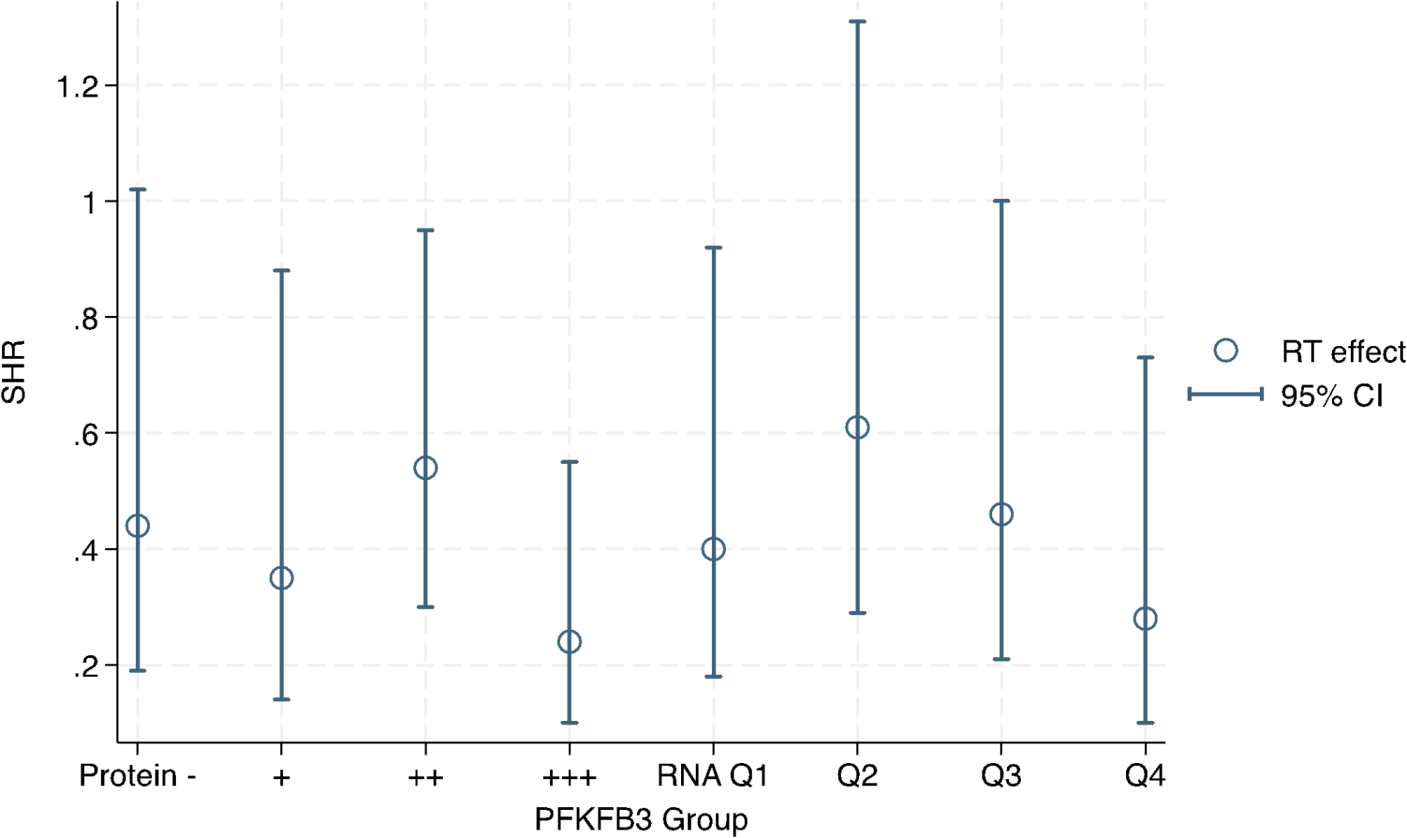
Subhazard ratios with 95% confidence interval for the effect of adjuvant radiotherapy on incidence of IBTR in different nuclear PFKFB3 protein and whole-cell RNA groups. Results from multivariable competing risks regression Also adjusting for interaction between PFKFB3 and radiotherapy, age, adjuvant systemic treatment, Nottingham grade, and subtype. CI – confidence interval, IBTR – ipsilateral breast tumor recurrence, Q – quartile, RT – radiotherapy, SHR – subhazard ratio

The next step was to evaluate PFKFB3 as a predictive marker of use for adjuvant radiotherapy. The effect of radiotherapy on the incidence of IBTR in relation to PFKFB3 levels was assessed in multivariable competing risks regression with an interaction term between PFKFB3 and radiotherapy. Age, adjuvant systemic therapy, Nottingham grade and tumor subtype was also included in the regression model. Results are presented in Fig. 3; Table 3. The evidence of an effect of radiotherapy was weak (*p* = 0.055) in the lowest protein group (SHR 0.44, 95% CI 0.19–1.02). The largest effect of radiotherapy was found in the groups with highest PFKFB3 protein group with SHR 0.24 (95% CI 0.10–0.55). Similar results were seen for RNA groups as there was no evidence (*p* = 0.21) for an effect of adjuvant radiotherapy in RNA Q2 (SHR 0.61, 95% CI 0.29–1.31) and the largest effect in Q4 (SHR 0.28, 95% CI 0.10–0.73). Our data suggested that tumors that highly expressed PFKFB3 might be more sensitive to radiotherapy.

However, there was no evidence of an interaction between PFKFB3 protein or RNA and effect of radiotherapy (p ranging from 0.32 to 0.81, Supplementary Table 3). To study whether PFKFB3 levels changed in tumors recurring after radiotherapy, the difference in histoscore was compared between IBTRs and their paired primary tumor. No association between radiotherapy and change in PFKFB3 was observed (*p* = 0.78). In short, we found insufficient evidence for PFKFB3 as a predictor of clinical use for adjuvant radiotherapy.

**Table 3:** Effect of adjuvant radiotherapy on incidence of IBTR in different PFKFB3 protein and RNA groups. Multivariable competing risks regression. IBTR is within 10 years

	SHR (95% CI)	*p*-value
**PFKFB3 Protein**		
-	0.44 (0.19–1.02)	0.055
+	0.35 (0.14–0.88)	0.026
++	0.54 (0.30–0.95)	0.034
+++	0.24 (0.10–0.55)	0.001
***PFKFB3 *** **RNA**		
Quartile 1	0.40 (0.18–0.92)	0.030
Quartile 2	0.61 (0.29–1.31)	0.21
Quartile 3	0.46 (0.21-1.00)	0.050
Quartile 4	0.28 (0.10–0.73)	0.009

Also adjusting for interaction between PFKFB3 and radiotherapy, age, adjuvant systemic treatment, Nottingham grade, and subtype. CI – confidence interval, SHR – subhazard ratio

### Differential gene expression and PFKFB3

**Fig. 4:**
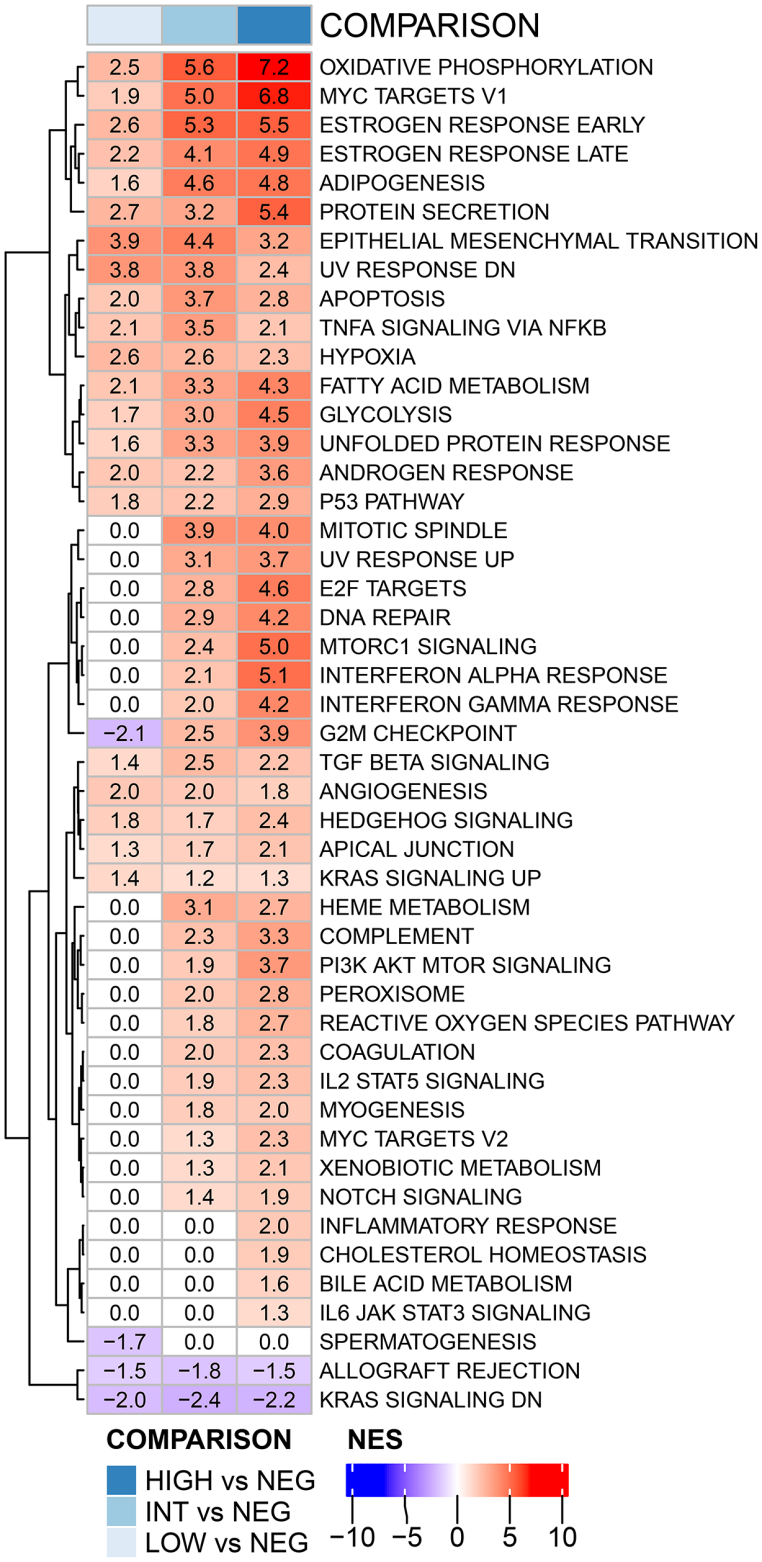
Gene set enrichment analysis (Hallmarks database), in the different PFKFB3 protein groups high/+++, int/++, low/+ compared to neg/-. NES – normalized enrichment score

To further assess the biological differences in tumors with varying PFKFB3 protein levels, differential expression analysis was performed. A total of 178 coding genes were differently expressed between protein-derived PFKFB3 lowest (-) and highest (+++) expression groups. Of these were 135 upregulated (e.g. NDUFAB1, TFF1, PYGL) and 43 downregulated (e.g. BMPRT1B, CSF1, RPL39L). GSEA showed that compared to the lowest protein group (-), 47 out of 50 Hallmarks pathways were enriched in one or several of the PFKFB3 positive protein groups (i.e. +, ++, or +++) over the PFKFB3 negative one (Fig. 4). Pathways related to metabolism showed a PFKFB3 level-dependent increase of enrichment, such as the oxidative phosphorylation, glycolysis, and fatty acid metabolism pathways. GSEA confirmed enrichment of the DNA repair and damage pathways p53, DNA repair and reactive oxygen species in tumors with high PFKFB3. The proliferation pathways MYC targets v1, G2M checkpoint and mTORC1 signaling were also PFKFB3 dependently enriched. The estrogen response pathways were highly enriched with increasing PFKFB3 protein groups, consistent with PFKFB3 being enriched in hormone positive tumor subtypes. The hypoxia pathway was enriched in all three groups compared to the lowest one but without a stepwise correlation, indicating that hypoxia is not an important source for PFKFB3 increase in this breast cancer subset. In conclusion, GSEA confirmed that PFKFB3 is significantly related to DNA repair, among other pathways, in breast cancer.

### Association between PFKFB3 protein and other markers

Next, we explored the associations between nuclear PFKFB3 protein and other biologically correlated proteins (Supplementary Table 4). Weak correlations were observed between nuclear PFKFB3 protein and the proliferation marker Ki67 (ρ = 0.19, *p* < 0.001), the transcription factor HIF-1α (ρ = 0.10, *p* = 0.001), the DNA damage marker γH2AX (ρ = 0.12, *p* < 0.001) and the anti-apoptotic protein p53 (ρ = 0.17, *p* < 0.001). Strong evidence for an association between PFKFB3 protein categories and micronuclei (indicator of chromosomal instability) occurrence was found, as the proportion of tumors with a large fraction (1–5%) of micronuclei gradually increased with increasing PFKFB3 protein levels (*p* < 0.001, Supplementary Table 5), but not for RNA levels (*p* = 0.37). This suggests that while PFKFB3 protein is related to DNA damage repair, cells with high levels of nuclear PFKFB3 protein are still experiencing chromosomal instability. No correlations were observed between nuclear PFKFB3 protein and the transcription factor pAkt or RAD51, a marker of HR repair. In summary, even though there are known biological associations between the proteins, no strong correlations were found in our dataset.

### Prognosis for IBTR and BCD

Next, we investigated the association between protein and RNA levels to IBTR as first event within 10 years and BCD within 15 years. Here, neither protein nor RNA levels were prognostic for IBTR or BCD in uni- or multivariable analyses (Table 4 and Supplementary Table 6). Further, there was no evidence for protein or RNA levels having an effect on the incidence of IBTR and BCD in any subtype. Cumulative incidence functions for IBTR depending on PFKFB3 levels and stratified by subtype is presented in Supplementary Figs. 3 and 4. In the light of these results, PFKFB3 does not appear to be useful prognostic marker in early-stage breast cancer.

**Table 4:** Nuclear PFKFB3 protein and RNA levels and incidence of IBTR and BCD. Multivariable competing risks regression. IBTR is within 10 years and BCD within 15 years after randomization

	Protein		RNA	
**IBTR**	SHR (95% CI)	*p*-value	SHR (95% CI)	*p*-value
**PFKFB3**				
- / Q1 (ref)	1		1	
+ / Q2	0.93 (0.52–1.67)	0.81	1.06 (0.63–1.78)	0.83
++ / Q3	1.20 (0.74–1.95)	0.45	1.07 (0.62–1.84)	0.82
+++ / Q4	0.95 (0.57–1.59)	0.84	0.90 (0.52–1.58)	0.72
**BCD**				
**PFKFB3**				
- / Q1 (ref)	1		1	
+ / Q2	1.30 (0.72–2.34)	0.38	0.90 (0.51–1.59)	0.72
++ / Q3	1.03 (0.61–1.74)	0.90	1.25 (0.71–2.20)	0.44
+++ / Q4	1.12 (0.65–1.94)	0.68	1.07 (0.60–1.90)	0.83

BCD - breast cancer death, CI – confidence interval, IBTR – ipsilateral breast tumor recurrence, Q – quartile, ref – reference, SHR – subhazard ratio

In IBTR analysis adjusting for age, adjuvant systemic treatment, Nottingham grade, and subtype

In BCD analysis adjusting for tumor size, Nottingham, grade, and subtype

## Discussion

In this large, randomized breast cancer trial tumors with the highest PFKFB3 levels showed the largest reduction of IBTR after adjuvant radiotherapy. However, PFKFB3 cannot predict the clinical use of adjuvant radiotherapy in breast cancer. To our knowledge, this is the first study that has investigated PFKFB3 as a treatment predictive marker in breast cancer. PFKFB3 could still be a target for radiosensitization and inhibitory treatment in general. *PFKFB3* RNA expression varied with tumor subtype, indicating that a future inhibitory treatment may be more effective in the Luminal A and HER2 positive tumors than the in triple negative and Luminal B subtype.

Even though PFKFB3 is related to DNA repair and that inhibition of it impairs homologous recombination repair after irradiation of cells, adjuvant radiotherapy more effectively reduced the incidence of IBTR in tumors with high levels PFKFB3. This indicates that tumors with enriched DNA repair systems responds to radiotherapy-induced DNA damage with apoptosis, as intended. In tumors with downregulated DNA repair systems, a subset of tumor cells may continue to live with this DNA damage, due to impaired DNA repair signaling. Another possible explanation to why tumors with high PFKFB3 seemed more sensitive to adjuvant radiotherapy is that they could have upregulated DNA repair system due to a large amount of endogenous DNA damage. This is supported by the fact that tumors with high nuclear PFKFB3 protein correlated to a higher degree of chromosomal instability and micronuclei.

We observed varying *PFKFB3* RNA levels in different subtypes, which was also supported in the GSEA as increased nuclear PFKFB3 protein levels correlated to enrichment of the estrogen response pathways. This could have implications in future treatment recommendations, as PFKFB3 inhibition in general could be more effective in the Luminal A and HER2 positive subtypes. The observation of higher levels of PFKFB3 in HER2 positive tumors is consistent with previous findings [4]. To our knowledge the fact that triple negative breast cancer shows decreased levels of *PFKFB3* RNA is new. These tumors are highly proliferative and could be suspected to show high PFKFB3 expression [26]. However, since estrogen and progesterone are transcriptional factors increasing PFKFB3 levels it is expected that tumors negative for both hormone receptors have less *PFKFB3* RNA [27, 28]. We did not observe the same association for nuclear PFKFB3 protein, suggesting that the nuclear levels PFKFB3 are independent of cytosol concentrations. Thus, using PFKFB3 inhibition as a radiosensitizer could potentially be equally effective among the different subtypes, as the DNA repair takes place in the nucleus. However, this is yet to be investigated.

In regards of PFKFB3 not being a prognostic marker in breast cancer, previous studies have shown the opposite. A retrospective analysis in HER2 positive breast cancers showed that high levels of *PFKFB3* mRNA correlated to shorter progression-free survival and distant metastasis-free survival [4]. In a recently published article, Kashyap et al. retrospectively saw that high *PFKFB3* expression correlated to reduced overall survival in breast cancer in general and in triple negative breast cancer, in particular [29]. Furthermore, a small study assessing the immunohistochemical staining of PFKFB3 in relation to overall survival and distant metastasis showed worse prognosis with increased protein levels [26]. The same was observed for overall survival and recurrence-free survival using gene expression of *PFKFB3*, in a larger subset of patients in the same article. In summary, previous studies have shown that primarily RNA levels but also protein levels of PFKFB3 are prognostic in breast cancer. We were unable to find the same association between PFKFB3 and prognosis, and this could be due to the lack of more aggressive tumor stages in our dataset. Another potential explanation is that few patients have received adjuvant treatment other than radiotherapy in our study, and that the prognostic effect of PFKFB3 could be potentiated by endocrine or anti-HER2 treatment.

To our knowledge, this is the first study to compare immunohistochemical staining of PFKFB3 protein to RNA levels. The immunohistochemical staining represents nuclear PFKFB3 protein, while the RNA levels are based on material from a whole tumor tissue punch. This will inevitably lead to discrepancies between nuclear protein and RNA levels and only a weak correlation between these was found. The discrepancy could also be affected by variations in degradation of protein and RNA as well as post-transcriptional regulation. These differences were later reflected in the in associations with clinicopathologically relevant variables, as nuclear protein was associated with tumor grade and RNA with tumor subtype. This was interpreted as the nuclear PFKFB3 protein levels reflecting nuclear events and RNA levels giving a more general image of tumoral activities.

## Conclusions

We conclude that in this patient material, high PFKFB3 was associated with a larger effect of adjuvant radiotherapy on incidence of IBTR but that PFKFB3 cannot reliably predict the clinical use of radiotherapy in breast cancer. *PFKFB3* expression differed with subtype, indicating that it could serve as a better predictive marker in in Luminal A and HER2 positive tumors compared to Luminal B and triple negative subtypes, but this is yet to be investigated.

## Electronic supplementary material

Below is the link to the electronic supplementary material.

Supplementary Material 1

## Data Availability

The gene expression data can be accessed at Gene Expression Omnibus (GSE119295, https://www.ncbi.nlm.nih.gov/geo/query/acc.cgi? acc=GSE119295).

## Abbreviations

BCD: Breast cancer death
CI: Confidence interval
ER: Estrogen receptor
GSEA: Gene set enrichment analysis
HER2: Human epidermal growth factor receptor 2
HIF-1α: Hypoxia-inducible factor 1α
HR: Homologous recombination
IBTR: Ipsilateral breast tumor recurrence
IQR: Interquartile range
mm: Millimeter
NES: Normalized enrichment score
PFKFB3: 6-phophofructo-2-kinase/fructose-2,6-bisphosphatase 3
PgR: Progesterone receptor
PT: Primary tumor
Q: Quartile
Ref: Reference
RT: Radiotherapy
SD: Standard deviation
SHR: Subhazard ratio
TMA: Tissue microarray
