# Supplementary material for: The potential radiosensitization target PFKFB3 is related to response to radiotherapy in SweBCG91RT: a randomized clinical trial with long-term follow-up
Source: BMC Cancer. 2025 Feb 28;25:374. doi: 10.1186/s12885-025-13703-1 (PMC11869729; doi:10.1186/s12885-025-13703-1)
Supplement: Supplementary file 1 — Supplementary Material 1 [file 12885_2025_13703_MOESM1_ESM.docx]

Supplement

Supplementary methods

Staining and scoring of the associated biomarkers used are described below.

*RAD51 and geminin*

A dual staining for RAD51 and geminin was performed to enable scoring of RAD51 foci only in cells in S-G2 phase. The immunohistochemical staining was performed on the fully automated instrument DISCOVERY ULTRA (Ventana Medical Systems Inc, Tucson, AZ, US) by using primary antibodies RAD51 (clone EPR4030-4, dilution 1:500 ab133534, Abcam, Cambridge, UK) and Geminin (poly, dilution 1:2000) I0802-I-AP, Proteintech, Rosemont, IL, US). Nuclear RAD51 staining alone was assessed as 0 (0%), 1 (< 5%), 2 (5-25%), 3 (26-50%), 4 (51-75 %), and 5 (> 75%). For the combined RAD51/geminin variable, only cores with at least 30 tumor cells positively stained for geminin were included. The fraction of cells with at least two nuclear RAD51 foci among geminin-positive cells were assessed using the abovementioned score of 0-5.

*Micronuclei*

Staining was performed using DAPI. Micronuclei were identified by the criteria: a close distance to the mother nucleus; within the same cytoplasm; similar intensity of staining as the mother cell nuclei; 1/3-1/16 the diameter of the nucleus; and a round to oval shape with a smooth perimeter suggesting a membrane. The fraction of micronuclei per 100 tumor cells was scored in categories of 0 (0%), 1 (< 1%), and 2 (1-5%). No tumor had a higher fraction than 5%.

*γH2AX*

Staining and scoring have previously been performed for a different project, using antibody #9718 (Cell Signaling Technology, Danvers, MA, US) with dilution 1:500. Fraction of nuclear staining was scored as 0 (0%), 1 (< 10%), 2 (10-50%), and 3 (> 50%) and intensity of scoring as 1 (Weak), 2 (Moderate), and 3 (Strong). A histoscore was created by multiplying fraction score with intensity score, generating values range 0-9. Cells with the morphological appearance of undergoing apoptosis were excluded.

*p53*

Staining and scoring have previously been performed for a different project, using the DO-1 clone from Santa Cruz (sc-126, Dallas, TX, US) with dilution 1:500. Fraction of nuclear staining was scored as exact values (0-100%) and intensity as 1 (Weak), 2 (Moderate), and 3 (Strong). A histoscore was created by multiplying fraction score with intensity score, generating values ranging from 0 to 300.

*Ki67*Staining and scoring have previously been performed for a different project, using the Ki67 antibody IR626 (clone MIB-1, DAKO, Agilent Technologies, Santa Clara, CA, US). The fraction of positively stained tumor cells (%) was scored according to the current guidelines using unweighted global assessment [1].

*HIF-1α*

Staining and scoring have previously been performed for a different project [2], using a monoclonal HIF-1α antibody (BD610959, 1:50, Becton Dickinson). Tumors were classified into categories of Negative (< 1% positive cells or 1-9% with weak intensity), Low (1-9% of cells with moderate or high intensity or > 10% of cells with weak intensity), or High (> 10% of cells with moderate or high intensity).

*pAkt*

Staining and scoring have previously been performed for a different project [3], using antibody pAkt-Ser473 (#4060, 1:10, Cell Signaling Technology, Danvers, MA, US). Tumors were classified into categories of 0 (< 10% stained nuclei, regardless of intensity), 1 (> 10% stained nuclei with a low staining intensity), and 2 (> 10% strongly stained nuclei).

*References*

1. Leung SCY, Nielsen TO, Zabaglo L, Arun I, Badve SS, Bane AL, et al. Analytical validation of a standardized scoring protocol for Ki67: phase 3 of an international multicenter collaboration. NPJ Breast Cancer. 2016;2:16014.

2. Tutzauer J, Sjostrom M, Holmberg E, Karlsson P, Killander F, Leeb-Lundberg LMF, et al. Breast cancer hypoxia in relation to prognosis and benefit from radiotherapy after breast-conserving surgery in a large, randomised trial with long-term follow-up. Br J Cancer. 2022;126(8):1145-56.

3. Sjostrom M, Veenstra C, Holmberg E, Karlsson P, Killander F, Malmstrom P, et al. Expression of HGF, pMet, and pAkt is related to benefit of radiotherapy after breast-conserving surgery: a long-term follow-up of the SweBCG91-RT randomised trial. Mol Oncol. 2020;14(11):2713-26.

Supplementary results

Supplementary table 1. Cutoffs for division of estrogen receptor, progesterone receptor, human epidermal growth factor 2 (HER2) and Ki67. Subtyping according to St Gallen International Breast Cancer Conference 2013.

|  | **Luminal A-like** | **Luminal B-like*** | **HER2 positive**** | **Triple negative** |
| --- | --- | --- | --- | --- |
| **Estrogen receptor** | > 1% | > 1% | Any | < 1% |
| **Progesterone receptor** | > 20% | < 20% | Any | < 1% |
| **HER2** | < 3+ and non-amplified | < 3+ and non-amplified | 3+ and/or amplified | < 3+ and non-amplified |
| **Ki67** | < 10% | > 10% | Any | Any |

*To be categorized as Luminal B-like, either low Progesterone receptor or high Ki67 was needed.
**Luminal and non-luminal HER2 positive were combined, due to group sizes.

Supplementary table 2. Given treatment in different PFKFB3 protein and RNA groups. Number (%).

|  | **PFKFB3 protein** | | | | |
| --- | --- | --- | --- | --- | --- |
|  | - | + | ++ | +++ | p |
| **Radiotherapy** |  |  |  |  |  |
| No radiotherapy | 110 (52%) | 99 (54%) | 160 (51%) | 133 (51%) |  |
| Radiotherapy | 100 (48%) | 84 (46%) | 154 (49%) | 130 (49%) | 0.55 |
| **Adjuvant systemic therapy** |  |  |  |  |  |
| No additional therapy | 197 (94%) | 167 (91%) | 287 (91%) | 239 (91%) |  |
| Endocrine therapy | 10 (5%) | 12 (7%) | 20 (6%) | 21 (8%) |  |
| Chemotherapy | 3 (1%) | 1 (1%) | 4 (1%) | 2 (1%) |  |
| Both | 0 (0%) | 3 (2%) | 3 (1%) | 1 (0%) | 0.64 |
|  | ***PFKFB3* RNA** | | | | |
|  | Q1 | Q2 | Q3 | Q4 | p |
| **Radiotherapy** |  |  |  |  |  |
| No radiotherapy | 100 (52%) | 106 (56%) | 93 (49%) | 104 (54%) |  |
| Radiotherapy | 92 (48%) | 85 (45%) | 98 (51%) | 87 (46%) | 0.55 |
| **Adjuvant systemic therapy** |  |  |  |  |  |
| No additional therapy | 178 (93%) | 174 (91%) | 180 (94%) | 171 (90%) |  |
| Endocrine therapy | 12 (6%) | 14 (7%) | 9 (4%) | 19 (9%) |  |
| Chemotherapy | 2 (1%) | 2 (1%) | 3 (2%) | 0 (0%) |  |
| Both | 0 (0%) | 1 (1%) | 0 (0%) | 2 (1%) | 0.24 |

Supplementary table 3. Multivariable competing risk regression analyses of effect of interaction between PFKFB3 levels (protein and RNA) and radiotherapy, on incidence of IBTR as first event within 10 years.

| **IBTR** | **Protein** | | **RNA** | |
| --- | --- | --- | --- | --- |
|  | SHR (95% CI) | p-value | SHR (95% CI) | p-value |
| **PFKFB3** |  |  |  |  |
| - / Q1(ref) | 1 |  | 1 |  |
| + / Q2 | 0.99 (0.49-2.00) | 0.98 | 0.93 (0.50-1.76) | 0.83 |
| ++ / Q3 | 1.12 (0.62-2.03) | 0.71 | 1.02 (0.52-1.97) | 0.96 |
| +++ / Q4 | 1.11 (0.61-2.04) | 0.73 | 0.98 (0.51-1.88) | 0.96 |
| **Radiotherapy** |  |  |  |  |
| No RT (ref) | 1 |  | 1 |  |
| Radiotherapy | 0.44 (0.19-1.02) | 0.055 | 0.40 (0.18-0.92) | 0.030 |
| **Interaction** |  |  |  |  |
| +/Q2# RT | 0.79 (0.22-2.79) | 0.71 | 1.53 (0.50-4.68) | 0.46 |
| ++/Q3 # RT | 1.23 (0.45-3.38) | 0.69 | 1.15 (0.37-3.55) | 0.81 |
| +++/Q4 # RT | 0.54 (0.16-1.80) | 0.32 | 0.69 (0.19-2.45) | 0.56 |

CI – confidence interval, IBTR – ipsilateral breast tumor recurrence, Q – quartile, ref – reference, RT – radiotherapy, SHR – subhazard ratio

Also adjusting for age, adjuvant systemic treatment, Nottingham grade, and subtype.

Supplementary table 4. Correlation matrix between associated markers and staining of nuclear PFKFB3 protein.

| **Associated markers** | **PFKFB3 Histoscore**  (Spearmans ρ) |
| --- | --- |
| Ki67 | 0.19* |
| HIF-1α | 0.10* |
| pAkt | 0.040^ns^ |
| RAD51, nuclear fraction | 0.055^ns^ |
| RAD51 foci in geminin+ cells | 0.082^ns^ |
| γH2AX | 0.12* |
| p53 | 0.17* |

* p < 0.001, ^ns^ non-significant
ρ 0-0.1 light red, ρ 0.1-0.2 dark red

Supplementary table 5. Comparison between nuclear PFKFB3 protein and whole-cell RNA levels and Micronuclei scoring categories. χ^2^ test.

|  | **Micronuclei** | | |  |
| --- | --- | --- | --- | --- |
|  | 0 | > 0 but < 1% | 1-5% |  |
| **PFKFB3 Protein** |  |  |  |  |
| - | 21 (38%) | 160 (21%) | 10 (12%) |  |
| + | 10 (18%) | 147 (19%) | 12 (14%) |  |
| ++ | 14 (25%) | 258 (33%) | 30 (36%) |  |
| +++ | 11 (20%) | 200 (26%) | 32 (38%) |  |
| p-value |  |  |  | 0.006 |
|  |  |  |  |  |
| **PFKFB3 RNA** |  |  |  |  |
| Q1 | 10 (33%) | 156 (25%) | 17 (23%) |  |
| Q2 | 4 (13%) | 157 (25%) | 23 (31%) |  |
| Q3 | 11 (37%) | 149 (24%) | 19 (25%) |  |
| Q4 | 5 (17%) | 156 (25%) | 16 (21%) |  |
| p-value |  |  |  | 0.37 |

Q - quartile

Supplementary table 6. Univariable competing risks regression analyses assessing effect PFKFB3 protein and RNA levels on incidence of IBTR as first event within 10 years and BCD within 15 years, respectively.

|  | **Protein** | | **RNA** | |
| --- | --- | --- | --- | --- |
|  | SHR (95% CI) | p-value | SHR (95% CI) | p-value |
| **IBTR** | | | | |
| **PFKFB3** |  |  |  |  |
| - / Q1 (ref) | 1 |  | 1 |  |
| + / Q2 | 0.83 (0.48-1.42)* | 0.50 | 1.12 (0.67-1.87) | 0.66 |
| ++ / Q3 | 1.09 (0.69-1.70) | 0.72 | 1.05 (0.63-1.78) | 0.84 |
| +++ / Q4 | 0.94 (0.58-1.52) | 0.81 | 0.93 (0.54-1.57) | 0.77 |
|  | | | | |
| **BCD** | | | | |
| **PFKFB3** |  |  |  |  |
| - / Q1 (ref) | 1 |  | 1 |  |
| + / Q2 | 1.09 (0.63-1.89) | 0.75 | 0.87 (0.50-1.51) | 0.61 |
| ++ / Q3 | 0.94 (0.58-1.53) | 0.81 | 1.13 (0.67-1.93) | 0.64 |
| +++ / Q4 | 1.05 (0.64-1.73) | 0.20 | 0.95 (0.55-1.65) | 0.87 |

* Non-proportional subhazard.

BCD - breast cancer death, CI – confidence interval, IBTR – ipsilateral breast tumor recurrence, Q – quartile, ref – reference, SHR – subhazard ratio


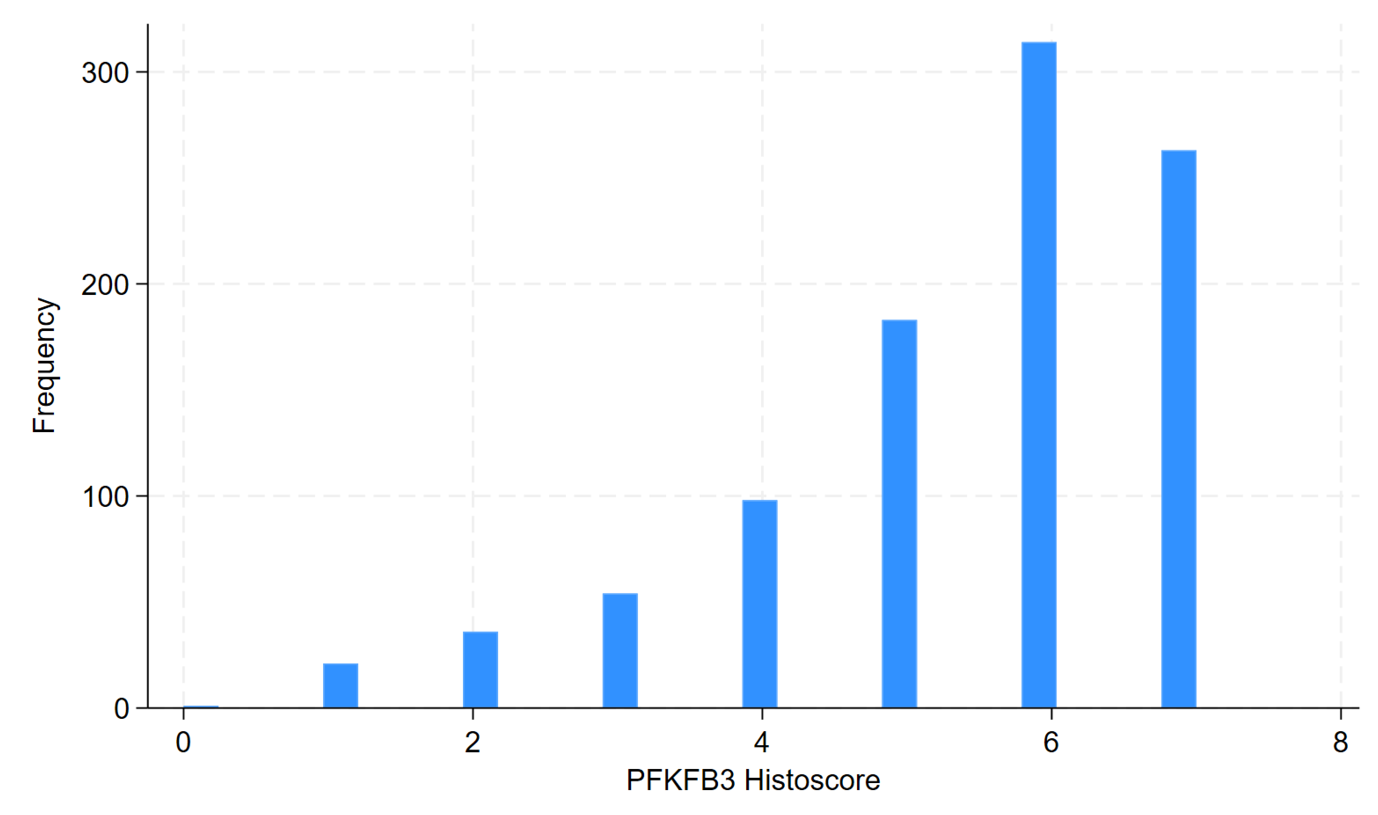
Supplementary figure 1. Distribution of histoscore of nuclear staining of PFKFB3 protein.


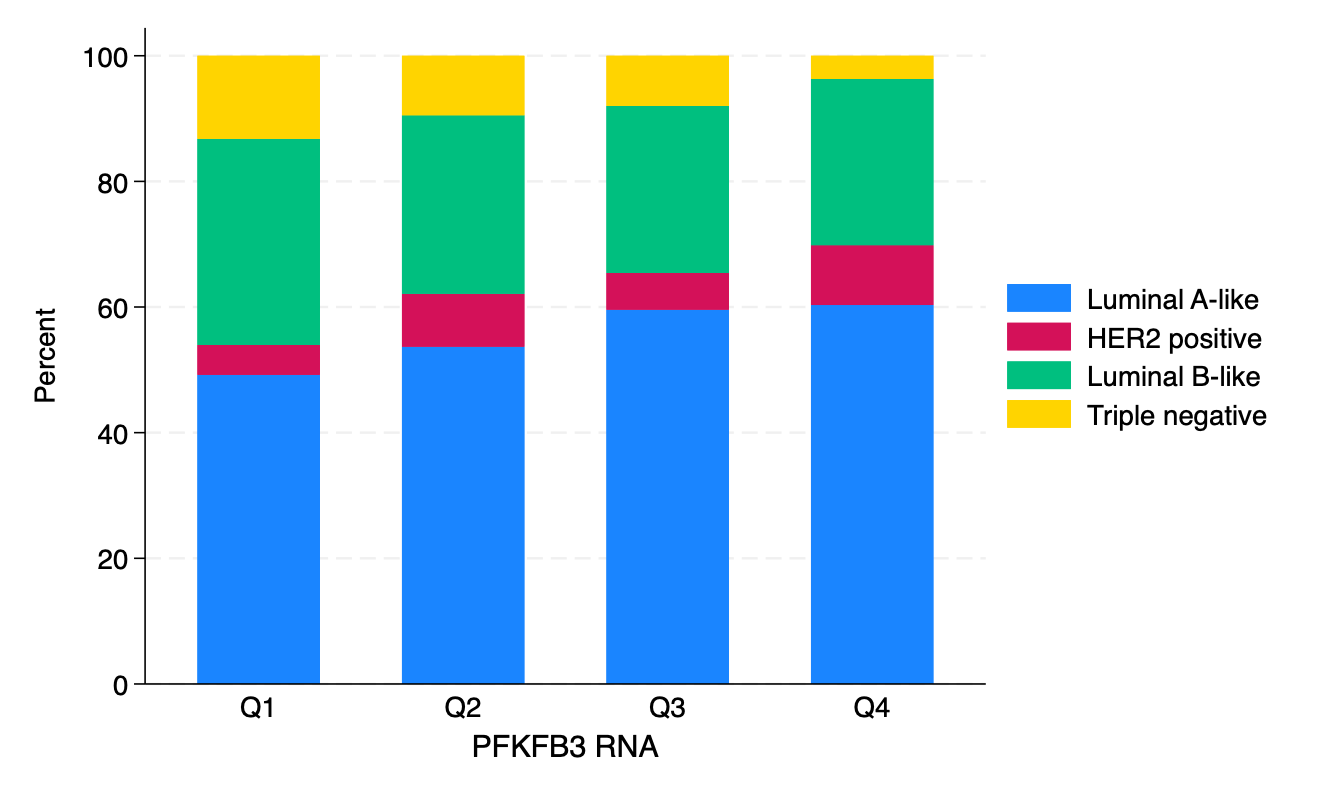
Supplementary figure 2. Box plot over *PFKFB3* RNA levels depending on tumor subtype.

HER2 - human epidermal growth factor 2. Q – Quartile.
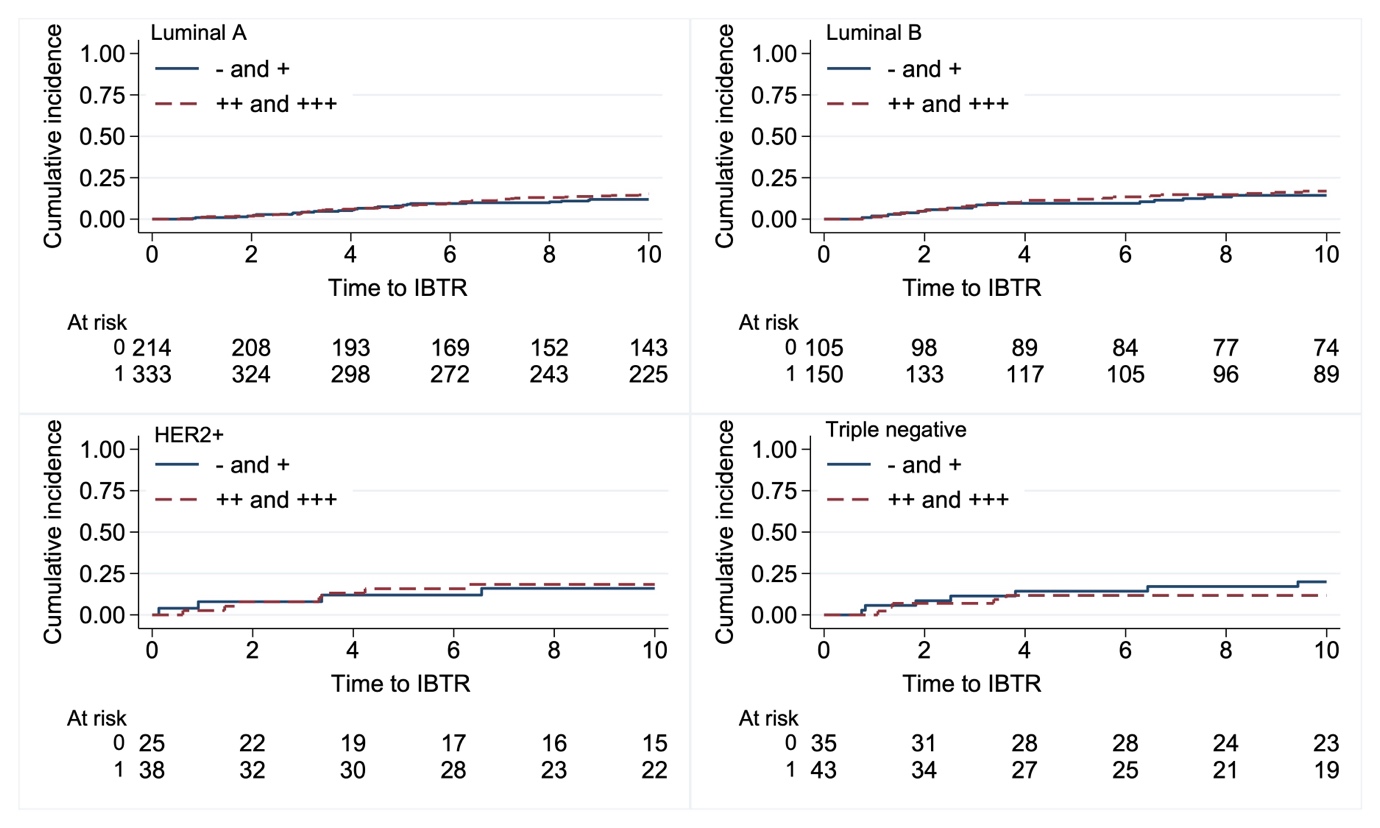


Supplementary figure 3. Cumulative incidence functions for IBTR as first event within 10 years, in different tumor subtypes and depending on PFKFB3 protein levels.

HER2 human epidermal growth factor 2, IBTR – ipsilateral breast tumor recurrence


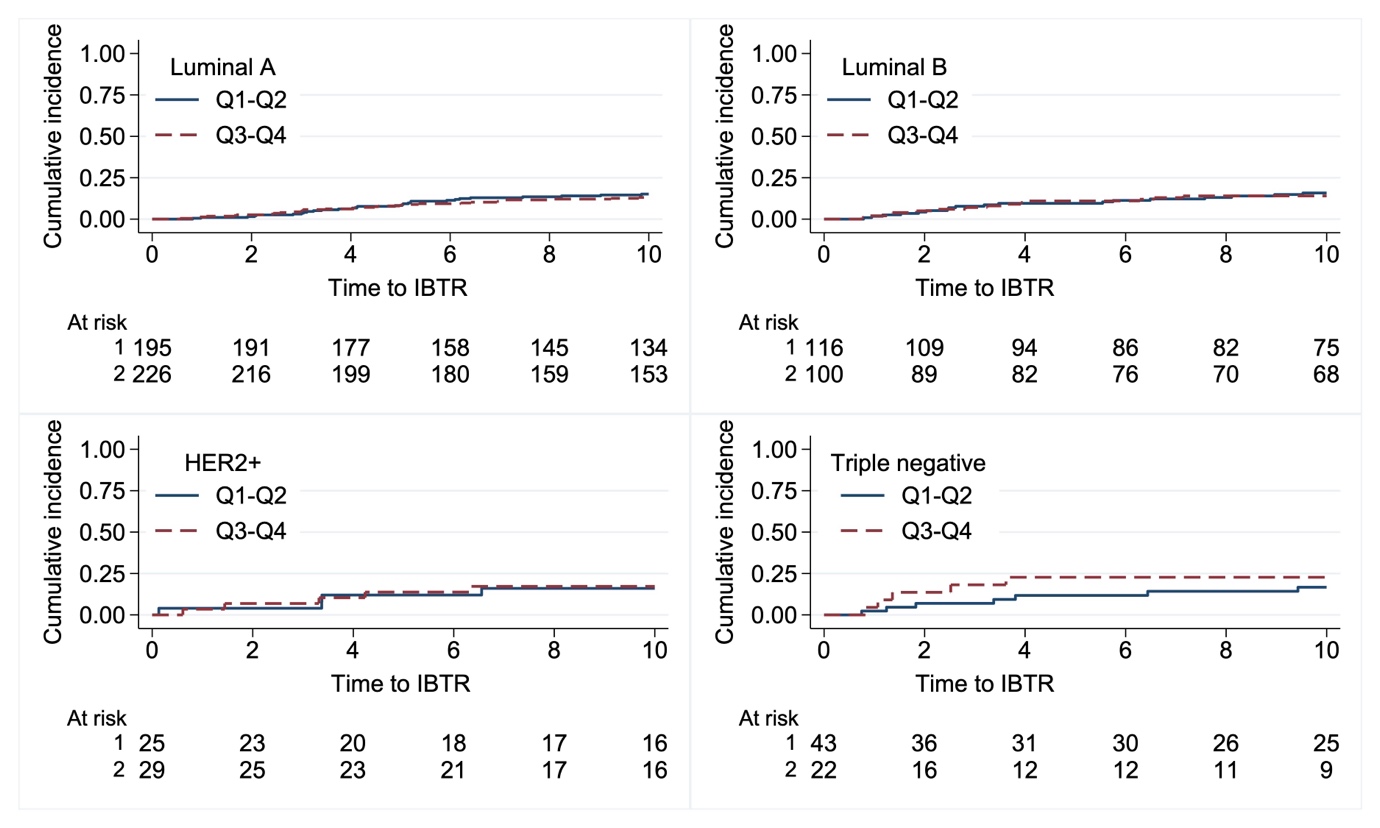


Supplementary figure 4. Cumulative incidence functions for IBTR as first event within 10 years, in different tumor subtypes and depending on *PFKFB3* RNA levels.

HER2 human epidermal growth factor 2, IBTR – ipsilateral breast tumor recurrence, Q - quartile
